# LC-MS/MS-Based Concurrent Quantification of Cannabidiol and Melatonin in Mouse Plasma to Elucidate Complex PK Interactions

**DOI:** 10.3390/pharmaceutics16121511

**Published:** 2024-11-25

**Authors:** Mengran Wang, Wenpeng Zhang, Xia Wu, Lingchao Wang, Cong Li, Chunyan Liu, Xiaomei Zhuang

**Affiliations:** 1 Beijing Institute of Pharmacology and Toxicology, Beijing 100850, China; wmr0212@outlook.com (M.W.); zhangwp@bmi.ac.cn (W.Z.); 15132816902@163.com (X.W.); wanglingchao624@163.com (L.W.); seancong1005@163.com (C.L.); 2 College of Pharmacy, North China University of Science and Technology, Tangshan 063210, China; chunyanliu@ncst.edu.cn

**Keywords:** melatonin, cannabidiol, LC-MS/MS, pharmacokinetics, complex drug–drug interactions

## Abstract

**Objective:** This study aimed to develop a quantitative analytical method for the simultaneous determination of cannabidiol (CBD) and melatonin (MT) in mouse plasma using the protein precipitation method coupled with LC-MS/MS. Additionally, this study sought to investigate the impact of CBD on the pharmacokinetics of MT in mice using this method. **Methods:** Mouse plasma samples were precipitated with acetonitrile and analyzed using a Kromasil 100-5-C8 (2.1 × 50 mm) column. Following a single administration, thirty male ICR mice were randomly assigned to five groups: MT 2 mg/kg intravenously (*i.v.*), MT 10 mg/kg orally (*p.o.*), MT + CBD (10 + 10) mg/kg *p.o.*, MT + CBD (10 + 40) mg/kg *p.o.*, and MT 10 mg/kg *p.o.* followed by CBD 2 mg/kg *i.v.* Pharmacokinetic parameters were calculated using a non-compartmental model and analyzed to investigate the interactions of CBD with MT. **Results:** The calibration curves for CBD and MT were linear over the range of 2 to 1000 ng/mL. Co-administration of a high dose of CBD (40 mg/kg) orally reduced the C_max_ of MT (10 mg/kg) to 57% of the control, while the area under the curve from 0.5 to 8 h (AUC_(0.5–8h)_) was 2.85-fold that of the MT-only group. When CBD (2 mg/kg) was administered intravenously alongside MT orally, the AUC_(0.5–8h)_ was 1.54 times that of MT given orally alone. The AUC of CBD was positively correlated with the AUC of the distribution and elimination phases of MT, while the C_max_ of CBD negatively correlated with the C_max_ of MT. **Conclusions:** The developed LC-MS/MS method is robust and suitable for pharmacokinetic studies involving CBD and MT. The in vivo effects of CBD on MT pharmacokinetics are complex. High oral doses of CBD inhibit both the intestinal absorption and metabolic clearance of MT, resulting in a more smooth PK profile.

## 1. Introduction

Insomnia not only degrades quality of life but also elevates the risk of a variety of health conditions. For those suffering from chronic insomnia, aside from cognitive behavioral therapy (CBT-I) [1], pharmacological interventions remain the predominant treatment approach. However, the commonly prescribed sleep aids are associated with undesirable side effects such as headache, dizziness, nausea, drowsiness, and in some cases, the potential for addiction. Melatonin (MT) has a wide range of pharmacological effects [2,3,4,5,6]. As a hormone that naturally regulates sleep, MT has increasingly been utilized as a dietary supplement or nutraceutical in recent years to complement the treatment of insomnia [7]. Research indicates that exogenous MT does not lead to dependency or a “hangover effect” and is associated with minimal side effects [8]. Nevertheless, MT undergoes extensive metabolism and rapid inactivation following oral administration, which diminishes its efficacy over time [9], a limitation that has impeded its development as a medicine.

In recent years, cannabidiol (CBD), a non-psychoactive compound, has emerged as a substance with significant medical potential [10,11,12,13,14,15,16,17]. A randomized, double-blind, controlled trial assessing the impact of oral cannabinoid and MT formulations on sleep disorders revealed that patients who received a combination of 5 mg MT, 15 mg CBD, and 15 mg cannabinol (CBN) experienced notable improvements in sleep quality compared to those who took 5 mg MT alone [18]. Additional research in rats and mice [19] evaluating the regulatory effects and mechanisms of a CBD-MT combination on circadian rhythm disturbances and insomnia also demonstrated that the combination possessed sedative and hypnotic properties, regulating sleep more effectively than MT alone.

It is recognized that MT is metabolized by hepatic enzymes CYP1A1, 1A2, and 1B1 to produce 6-hydroxymelatonin in humans [20,21,22]. CBD, on the other hand, is primarily metabolized by CYP2C9 and CYP2C19 to yield 7-hydroxy-CBD in humans [23]. Studies have also discovered that CBD can inhibit the activity of CYP1A1/2 [24,25,26]. The relationship between the sleep-enhancing effects of the CBD–MT combination and potential drug interactions mediated by CYP1A1/2 has yet to be explored.

Although benefits of the combination have been published earlier, in the absence of PK results, the underlying cause remains unknown. It is uncertain whether the efficacy outcomes stem from alterations in the concentrations of MT or CBD as a result of PK interactions or the synergistic effect of the drug’s efficacy. The objective of this study is to develop a LC-MS/MS method for the simultaneous detection of CBD and MT in mouse plasma. Furthermore, the study seeks to preliminarily investigate the pharmacokinetic profile of MT when co-administered with varying doses and administration routes of CBD in mice. This research aims to offer a scientific foundation for the development of a CBD–MT compound as a novel therapeutic agent.

## 2. Materials and Methods

### 2.1. Main Instruments

The LC-MS/MS instrument utilized was a Shimadzu LC-MS-8060 Triple quadrupole liquid chromatography mass spectrometer (Shimadzu, Kyoto, Japan). A FRESCO21 high-speed refrigerated centrifuge was purchased from Thermo (Waltham, MA, USA). A T-214 electronic analytical balance was purchased from Denver Instrument (Denver, CO, USA).

### 2.2. Animals

CD-1 mice (ICR mice, male, age: 42–48 days, weighting 30–40 g) were purchased from Beijing Vital River Laboratory Animal Technology Co., Ltd. (Beijing, China). All animal experiments conducted were in compliance with and approved by the Experimental Animal Ethics Committee of the Beijing Institute of Pharmacology and Toxicology (IACUC-DWZX-2024-505).

### 2.3. Chemicals

Melatonin (purity: 98%) was purchased from Beijing Solaibao Technology Co., Ltd. (Beijing, China). Melatonin D4 (Lot: C14347624) was obtained from Shanghai Maclin Biochemical Technology Co., Ltd. (Shanghai, China). Cannabidiol (purity: 98%) was sourced from Wuhan Zhongbiao Technology Co., Ltd. (Wuhan, China). Dimethyl sulfoxide (DMSO, purity: >99.7%) was obtained from Sigma-Aldrich (St. Louis, MO, USA). HPLC-grade acetonitrile and methanol were supplied by Thermo Fisher Scientific (Waltham, MA, USA). HPLC-grade formic acid was purchased from J&K Scientific (Beijing, China). Purified water was purchased from Hangzhou Wahaha Group Co., Ltd. (Hangzhou, China). Polyoxymethylene hydrogenated castor oil (purity: EL-40) was purchased from Shanghai Yuanye Biotechnology Co., Ltd. (Shanghai, China).

### 2.4. Preparation of Stock and Working Solutions

CBD and MT along with MT-D4 standards, were each accurately weighed to 10 mg and dissolved with DMSO to a final volume of 1 mL, separately, to prepare a stock solution with a mass concentration of 10 mg/mL. For analytical purposes, the stock solutions were diluted stepwise with acetonitrile to prepare a series of reference solutions at varying concentrations and an internal standard (IS) solution containing MT-D4 at a concentration of 10 ng/mL (MT-D4 is the internal standard of CBD and MT).

### 2.5. Sample Preparing

Plasma samples were processed using protein precipitation with acetonitrile. To each 10 μL aliquot of mouse plasma, 10 μL of blank acetonitrile was added, followed by the addition of 80 μL of acetonitrile containing the internal standard MT-D4 at a concentration of 10 ng/mL. The mixture was vortexed for 1 min and then centrifuged at 4 °C at 14,000× *g* for 10 min. A 70 μL aliquot of the supernatant was then taken for LC-MS/MS analysis.

### 2.6. Establishment of LC-MS/MS Methodology

**Figure 1:**
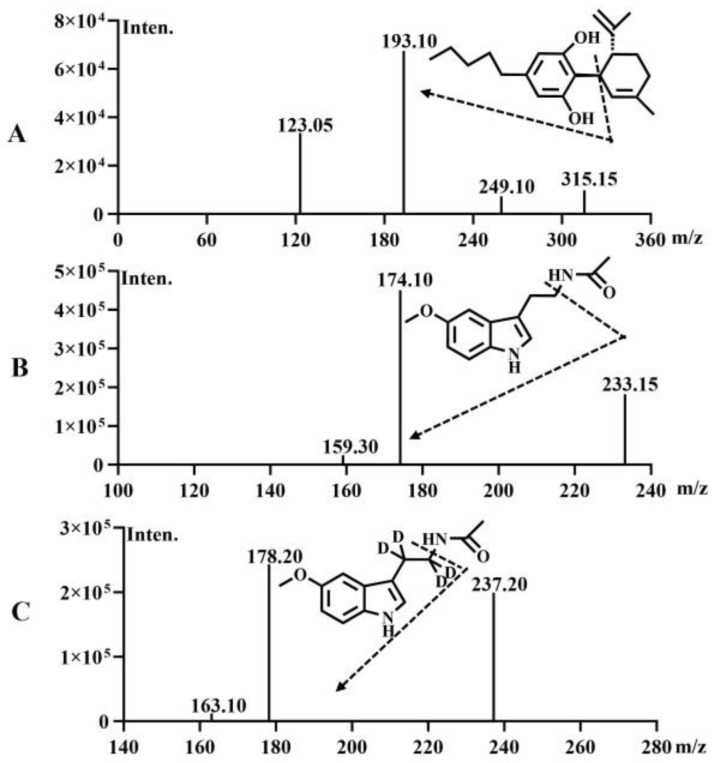
Characteristic mass spectrometry of CBD (**A**), MT (**B**) and MT-D4 (**C**). CBD: cannabidiol; MT: melatonin; MT-D4: melatonin-D4.

Mass Spectrometry Method: The drying gas flow rate was set at 10 L/min, the heating gas flow rate at 10 L/min, and the collision gas pressure at 270 kPa. The ion source temperature was maintained at 300 °C, the solvent tube temperature at 250 °C, and the heating module temperature at 400 °C. Using LC-MS/MS with an ESI ion source in positive ion scanning mode and MRM mode, the MRM transitions for CBD, MT and MT-D4 were set at 315.15–193.10, 233.15–174.10, and 237.20–178.20, respectively. The characteristic mass spectrometry ion chromatograms of CBD, MT, and MTD4 are depicted in Figure 1.

Chromatographic method: A Kromasil 100-5-C8 (2.1 × 50 mm) column was utilized to separate the analytes of interest. The mobile phases consisted of methanol (containing 0.1% formic acid) and water (containing 0.1% formic acid and 5 mM ammonium acetate). The gradient elution program was as follows: 0.0–0.5 min, 5% B; 0.5–1.5 min, 5–95% B; 1.5–3.0 min, 95% B; 3.0–3.1 min, 95–5% B; 3.1–4.0 min, 5% B. The flow rate was maintained at 0.5 mL/min, and the column temperature was kept at 40 °C. The injection volume for each analysis was 5 μL.

### 2.7. Method Validation

The quantification validation process was carried out according to the ICH-M10 guidelines (European Medicines Agency, EMA).

#### 2.7.1. Selectivity and Specificity

Double-blank (DB) mouse plasma samples, which contained neither the test substances nor the internal standard, were prepared. Mouse blank plasma was spiked with the internal standard and the test substances (10 μL of blank mouse plasma was taken, and 10 μL of either blank acetonitrile or a mixed working solution containing 100 ng/mL CBD and MT was added). Additionally, actual samples were collected after administration (plasma samples were obtained 15 min after intragastric administration of CBD + MT (10 + 10) mg/kg to the mice). Following protein precipitation with acetonitrile, the above plasma samples were analyzed using LC-MS/MS to determine the chromatographic characteristics of CBD, MT, and MT-D4.

#### 2.7.2. Preparation of Calibration and QC Samples

Blank mouse plasma (10 μL) was spiked with a mixed working solution containing CBD and MT to achieve final concentrations of 2, 5, 10, 50, 100, 200, 500, and 1000 ng/mL for each analyte (with the internal standard MT-D4 at a concentration of 10 ng/mL). Double blank plasma samples (DB) and plasma samples containing only the internal standard (CB) were also prepared. Lower limit of quantification (LLOQ) and quality control (QC) samples, including low QC (LQC), medium QC (MQC), and high QC (HQC), were prepared with final concentrations of CBD and MT in mouse plasma of 2, 4, 80, and 800 ng/mL.

#### 2.7.3. Accuracy and Precision

Quality control samples of CBD and MT at LLOQ, LQC, MQC, and HQC levels (2, 4, 80, 800 ng/mL) in mouse plasma were prepared in triplicate (*n* = 6). The accuracy and precision of these QC samples were assessed across three separate batches (*n* = 18), concurrently with the standard curve. The precision (relative standard deviation, RSD) and accuracy of the QC samples were calculated to evaluate the method’s performance.

#### 2.7.4. Extraction Recovery and Matrix Effect

Briefly, 10 μL of blank plasma from six different mice was aliquoted, to which 10 μL of a mixed working solution containing CBD and MT was added, followed by the addition of 80 μL of acetonitrile containing the internal standard MT-D4 at a concentration of 10 ng/mL. This mixture was used to prepare LQC and HQC samples (*n* = 6). After protein precipitation, the peak areas of CBD, MT, and the internal standard MT-D4 were measured using LC-MS/MS. To assess the matrix effect, the plasma was replaced with pure water, and the sample preparation and pretreatment were conducted identically to the plasma samples. The peak areas of CBD and MT, as well as the internal standard MT-D4, were then detected, with the ratio of the peak area of the plasma sample to that of the pure water sample representing the matrix effect.

Blank plasma from six different mice was pooled in equal parts. An aliquot of 10 μL of this pooled plasma was taken, and 10 μL of the mixed working solution containing CBD and MT was added either before or after protein precipitation to prepare LQC, MQC, and HQC samples (*n* = 6). The peak areas were detected by LC-MS/MS, and the ratio of the peak area before and after protein precipitation was defined as the method recovery.

#### 2.7.5. Stability

Quality control plasma samples of LQC, MQC, and HQC for mice (*n* = 3) were prepared, and the stability of CBD and MT were investigated after the samples were stored in LC-MS/MS storage for 24 h. Mouse plasma was stored at room temperature for 4 h, 4 °C for 24 h and frozen and thawed for 3 times, −40 °C for 7 days, or −40 °C for 21 days.

#### 2.7.6. Dilution Linearity

Mouse plasma samples (*n* = 6) containing CBD and MT at a concentration of 5000 ng/mL were prepared. The dilution linearity of mouse plasma was verified by conducting a 100-fold dilution with blank mouse plasma to achieve a final concentration of 50 ng/mL.

### 2.8. ICR Mouse Pharmacokinetic Studies

Thirty ICR male mice were randomly divided into 5 groups, each consisting of six mice, and each group received a single dose as follows: the MT 2 mg/kg intravenous (*i.v.*) group, the MT 10 mg/kg oral (*p.o.*) group, the MT + CBD (10 + 10) mg/kg *p.o.* group, the MT + CBD (10 + 40) mg/kg *p.o.* group, and the MT 10 mg/kg *p.o.* + CBD 2 mg/kg *i.v.* group. The volume of all administrations was 5 mL/kg, and all drugs were dissolved in saline with 5% castor oil. Before oral administration, mice fasted for 12 h and drank water freely. Every dose was given between 8:00 and 9:00 a.m., and the mice showed no abnormal behavior after administration. Sampling time points were established as follows: the MT *i.v.* group: 0.033, 0.083, 0.25, 0.5, 0.75, 1, 2, 4, and 6 h; other groups: 0.083, 0.25, 0.5, 0.75, 1, 2, 4, 6, and 8 h. Approximately 30 μL of blood was collected via intravenous puncture into heparinized anticoagulant tubes and immediately placed on ice. Whole blood samples were centrifuged at 4 °C, 5000× *g* for 10 min within 1 h, and the plasma was harvested and stored at −40 °C in a refrigerator for subsequent analysis. All collected samples were analyzed within 21 days. The dosage was selected according to previous studies [19], and the blood collection points and duration were determined according to the preliminary experiment to capture the whole PK profile.

### 2.9. Data Analysis and Statistical Processing

GraphPad Prism 8 software (San Diego, CA, USA) was utilized to generate graphical representations of the data. Major pharmacokinetic parameters, including the t_max_, C_max_, AUC, half-life (t_1/2_), clearance rate (CL), and volume of distribution (V) for each drug in the mice, were calculated using WinNonlin 8.1 software (Princeton, NJ, USA). These calculations were based on non-compartmental analysis. Non-compartmental analysis is a model-independent approach that estimates pharmacokinetic parameters directly from the observed plasma concentration–time curve. The bioavailability (F) was calculated using the following formula:


(1) $$F=\frac{{\mathrm{AUC}}_{\left(0–\infty ,\;\mathrm{extravascular}\right)}\times {\mathrm{Dose}}_{i.v.}}{{\mathrm{AUC}}_{\left(0–\infty ,\;i.v.\right)}\times {\mathrm{Dose}}_{\mathrm{extravascular}}}\times 100\%$$

Statistical analyses were performed using IBM SPSS Statistics 26. The experimental data were expressed as mean ± standard deviation (SD). Initially, the Shapiro–Wilk test was employed to assess the normality of the data distribution. For comparing differences among multiple groups, a one-way ANOVA was applied to determine if there were significant differences between the group means. When comparing two groups of data, an independent samples *t*-test was utilized for statistical analysis. This analysis mainly focused on the alterations of the in vivo exposure of MT after co-administration with CBD across different dosages and routes. A *p*-value less than 0.05 was considered to indicate a statistically significant difference, while a *p*-value less than 0.01 was considered to represent a highly significant difference. Linear regression analyses were performed on the AUC_(0–8h)_ of CBD and the AUC of MT, as well as the C_max_ of CBD and MT to explore potential correlations.

## 3. Results

### 3.1. Methodological Validation

#### 3.1.1. Specificity and Selectivity

**Figure 2:**
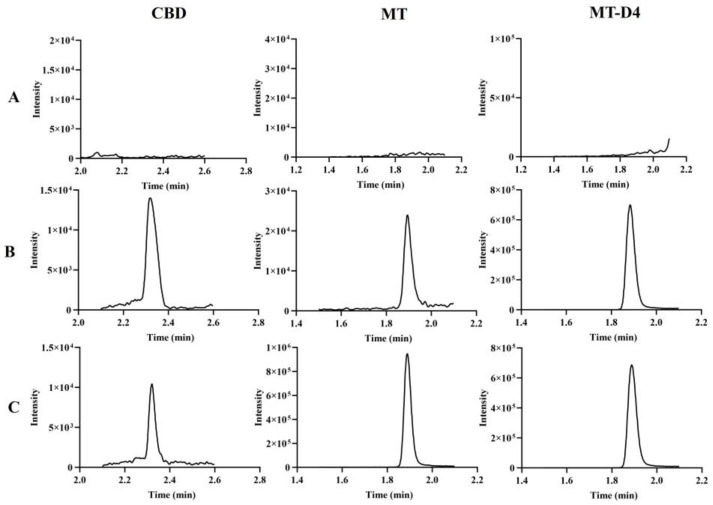
Representative chromatograms of CBD, MT, and MT-D4 in mice plasma. (**A**): Mouse blank plasma; (**B**): Mouse blank plasma spiked with a mixture of CBD + MT at LLOQ concentration (2 ng/mL); (**C**): Actual plasma sample in mouse obtained at 5 min after the administration of MT + CBD (10 + 10) mg/kg. CBD: cannabidiol; MT: melatonin; MT-D4: melatonin-D4.

Under the aforementioned detection conditions, all the signal-to-noise ratios (S/N) for the LLOQ levels of CBD, MT, and MT-D4 in mouse plasma exceeded 5. The chromatograms for blank plasma; blank plasma spiked with CBD, MT, and MT-D4 working solution; and plasma samples from mice post-administration are presented in Figure 2. The respective retention times for CBD, MT, and MT-D4 in mouse plasma were 2.31 min, 1.88 min, and 1.88 min. There was no interference from endogenous substances in mouse plasma for CBD, MT, and MT-D4.

#### 3.1.2. Linear Range

The calibration curves for CBD and MT were linear over the concentration range of 2 to 1000 ng/mL. The lowest limit of quantification was established at 2 ng/mL.

#### 3.1.3. Accuracy and Precision

The accuracy and precision data for CBD and MT in mouse plasma are summarized in Table 1. The inter- and intra-batch accuracies for the LLOQ were within the range of 87.55% to 113.37%, with a precision (relative standard deviation, RSD) of less than 8.7%. Similarly, the inter- and intra-batch accuracies for the QC samples were within the range of 94.08% to 112.52%, with a precision of less than 5.8%. These results indicate that the developed methodology is robust.

#### 3.1.4. Matrix Effects and Method Recoveries

The recoveries of CBD, MT, and MT-D4 in mouse plasma exceeded 90.43%. The matrix effects of CBD, MT, and the internal standard MT-D4 in mice plasma ranged from 99.21% to 109.94%, suggesting that there were no significant matrix effects. The detailed data are presented in Table 2, and all aspects of the methodology complied with the necessary standards.

#### 3.1.5. Stability

After sample treatment, the samples were stored for 24 h. Mouse plasma was stored at room temperature for 4 h, 4 °C for 24 h and frozen and thawed repeatedly for 3 times, stored at −40 °C for 7 days, or stored at −40 °C for 21 days. These samples were compared with the current plasma samples with the same concentration, and the relative concentrations of stable samples were obtained. The accuracy ranges from 88.67% to 112.63%. There were no significant concentration differences for CBD and MT stored in plasma under the aforementioned conditions. The results indicated that CBD and MT in mouse plasma samples remained stable under the corresponding storage conditions, which could support pharmacokinetic studies. The detailed data are displayed in Table 3.

#### 3.1.6. Dilution Integrity

Preliminary experimental results showed that the concentrations of CBD and MT exceeded the upper limit of quantification. Therefore, the plasma samples containing CBD and MT were diluted 100-fold with blank mouse plasma to a concentration of 50 ng/mL. The results demonstrated that the accuracy of the diluted samples was within the range of −6.0% to 10.2%, which meets the requirements for the quantification of plasma samples with higher drug concentrations after dilution. In the formal experiment, the plasma samples collected at 0.033 and 0.083 h were diluted for quantitative analysis since their concentrations exceeded the upper detection limit. The detailed results are presented in Table 4.

### 3.2. Pharmacokinetic Results in ICR Mice

#### 3.2.1. Pharmacokinetic Characteristics of MT in Mice

**Figure 3:**
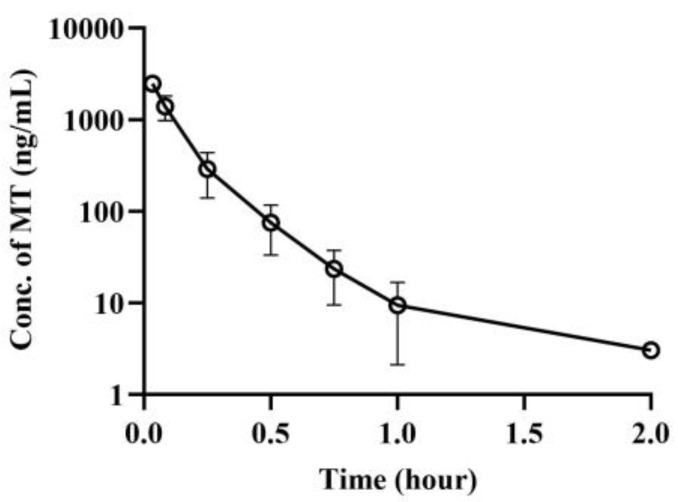
Plasma concentration–time curves of MT after *i.v.* administration of MT in mice (mean ± SD, *n* = 6). Plasma concentration–time curves of MT after *i.v.* administration of MT 2 mg/kg in mice. MT: melatonin.

The dose of MT (10 mg/kg via oral administration) in our pharmacokinetic (PK) study is consistent with the efficacy results [19]. For CBD, the dose of 40 mg/kg was selected based on our preliminary study and in combination with references where it was reported that a combination of 5 mg MT and 15 mg CBD led to notable improvements in sleep quality compared to those who took 5 mg MT alone. The plasma concentration–time curves of MT following intravenous injection of 2 mg/kg MT alone in mice are depicted in Figure 3. The corresponding pharmacokinetic parameters are displayed in Table 5. Upon intravenous administration of MT, the apparent volume of distribution (0.604 ± 0.067 L/kg) was found to be larger than the plasma volume (0.05 L/kg), and the clearance rate (5.21 ± 1.09 L/h/kg) was comparable to the liver blood flow (5.4 L/h/kg).

**Figure 4:**
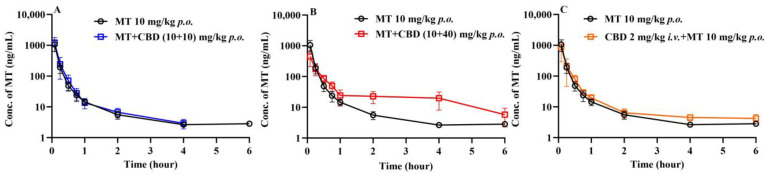
Plasma concentration–time curves of MT post different routes of administration in mice (mean ± SD, *n* = 6). Plasma concentration–time curves of MT after oral administration of MT 10 mg/kg and MT + CBD (10 + 10) mg/kg (**A**); Plasma concentration–time curves of MT after oral administration of MT 10 mg/kg and MT + CBD (10 + 40) mg/kg (**B**); Plasma concentration–time curves of MT after oral administration of MT 10 mg/kg and *i.v.* administration of CBD 2 mg/kg + oral administration of MT 10 mg/kg (**C**). CBD: cannabidiol; MT: melatonin.

The plasma concentration–time curves of MT after oral administration of MT 10 mg/kg are displayed in Figure 4. When 10 mg/kg of MT was administered orally alone, the mice exhibited rapid absorption, followed by swift clearance, resulting in a short residence time in vivo. The bioavailability of MT was determined to be 10.7%.

#### 3.2.2. Effects of CBD on the Pharmacokinetics of MT in Mice

**Figure 5:**
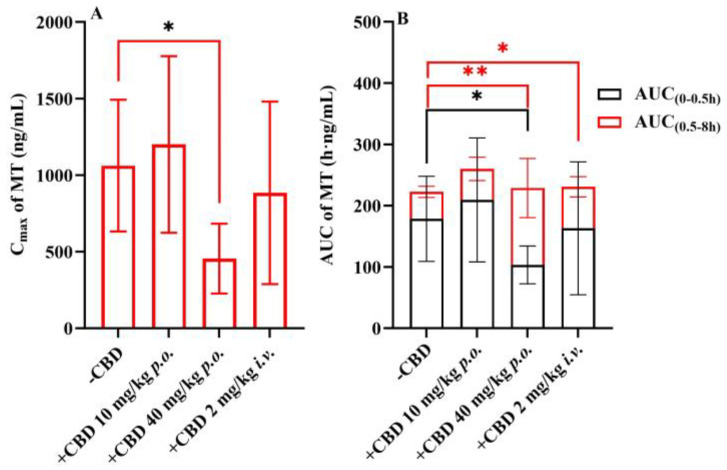
Comparison of C_max_ and AUC of MT in different groups (mean ± SD, *n* = 6). Comparison of C_max_ (**A**) and AUC (**B**) of MT in different groups after *p.o.* administration of MT 10 mg/kg, MT + CBD (10 + 10) mg/kg, MT + CBD (10 + 40) mg/kg, or *i.v.* CBD 2 mg/kg + *p.o.* administration of MT 10 mg/kg. (*: *p* < 0.05, statistically difference, **: *p* < 0.01, statistically significant difference, compared with MT 10 mg/kg group.) CBD: cannabidiol; MT: melatonin.

**Figure 6:**
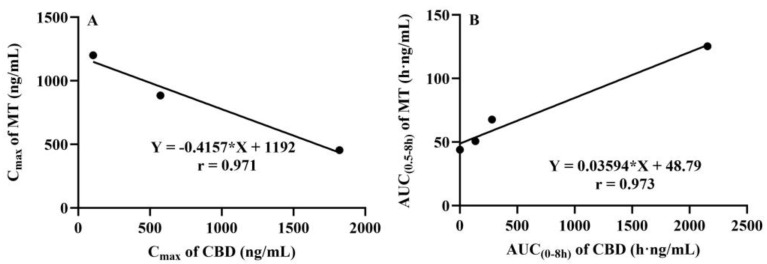
Correlation analysis between C_max_ of MT and CBD; correlation analysis between AUC_(0.5–8h)_ of MT and AUC_(0–8h)_ of CBD. Linear regression was conducted with C_max_ of CBD as the horizontal axis and C_max_ of MT as the vertical axis (**A**). Another linear regression was conducted with AUC_(0–8h)_ of CBD as the horizontal axis and AUC_(0.5–8h)_ of MT as the vertical axis (**B**). CBD: cannabidiol; MT: melatonin. *: multiply.

The plasma concentration–time curves of MT following the co-administration of CBD and oral MT via different routes are depicted in Figure 4. The corresponding pharmacokinetic parameters are presented in Table 5. The results indicated that the most significant alterations in the plasma concentration–time curves of MT were observed when MT was administered orally with a high dose of CBD (40 mg/kg). It is interesting to find that the absorption phase and the elimination phase exhibited opposite trends, resulting in a smoother curve. The outcomes of the C_max_ and AUC analyses are illustrated in Figure 5, which demonstrate that the oral administration of a high dose of CBD (40 mg/kg) considerably reduced the C_max_ of MT to 57%. The AUC_(0–0.5h)_, indicative of the absorption phase exposure, was also significantly diminished, whereas the AUC_(0.5–8h)_, representing the exposure during the distribution and elimination phases, increased to 2.85 times. However, the change in AUC_(0–∞)_, reflecting overall exposure, was not substantial. Subsequently, following the concurrent intravenous administration of CBD (2 mg/kg), the C_max_ of MT decreased to a lesser extent, but the AUC_(0.5–8h)_ was 1.54 times that of the oral group alone. The correlation analysis between the AUC of MT and the AUC_(0–8h)_ of CBD is displayed in Figure 6, revealing that the AUC_(0.5–8h)_ of MT has a good correlation with the AUC_(0–8h)_ of CBD (r = 0.973). The C_max_ of CBD negatively correlated with the C_max_ of MT (r = 0.971).

**Table 5:** Pharmacokinetic parameters of MT after administration of MT with or without CBD in mice (mean ± SD, *n* = 6).

Parameters	Unit	*i.v.* (mg/kg)	CBD 2 *i.v.* + MT 10 *p.o.*	*p.o.* (mg/kg)		
		MT 2		MT 10	MT + CBD (10 + 10)	MT + CBD (10 + 40)
t_1/2_	h	0.18 ± 0.07	0.19 ± 0.04	0.16 ± 0.02	0.16 ± 0.01	0.23 ± 0.03
T_max_	h	/	0.08 ± 0.00	0.08 ± 0.00	0.08 ± 0.00	0.11 ± 0.07
C_max_	ng/mL	/	884.1 ± 596.1	1061.9 ± 430.0	1200.2 ± 576.0	454.9 ± 227.6 *
AUC_(0–0.5h)_	h × ng/mL	/	163.1 ± 108.5	178.54 ± 69.38	209.3 ± 101.26	103.3 ± 30.9 *
AUC_(0.5–8h)_	h × ng/mL	/	67.64 ± 16.57 *	43.94 ± 9.11	50.63 ± 19.06	125.3 ± 48.15 **
AUC_(0–8h)_	h × ng/mL	402.1 ± 117.1	210.8 ± 96.5	210.3 ± 71.0	242.2 ± 103.7	207.2 ± 66.8
AUC_(0–∞)_	h × ng/mL	403.5 ± 117.2	218.6 ± 94.2	215.4 ± 69.8	247.2 ± 101.6	242.8 ± 71.6
MRT_(0–t)_	h	0.12 ± 0.02	0.65 ± 0.37	0.36 ± 0.15	0.33 ± 0.16	1.14 ± 0.43
V_z_	L/kg	1.26 ± 0.12	/	/	/	/
V_ss_	L/kg	0.60 ± 0.07	/	/	/	/
CL	L/h/kg	5.21 ± 1.09	/	/	/	/
F	%	/	/	10.7	/	/

*: *p* < 0.05, statistically difference, **: *p* < 0.01, statistically significant difference, compared to MT 10 mg/kg alone group. /: Not available. CBD: cannabidiol; MT: melatonin.

#### 3.2.3. Pharmacokinetic Characteristic of CBD in Mice

**Figure 7:**
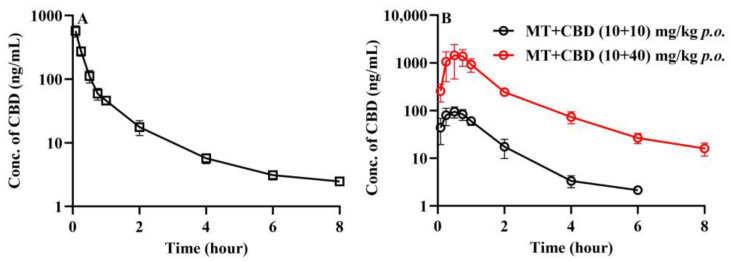
Plasma concentration–time curves of CBD after administration of CBD in mice (mean ± SD, *n* = 6). Plasma concentration–time curves of CBD after intravenous administration of CBD 2 mg/kg + oral administration of MT 10 mg/kg in mice (**A**); plasma concentration–time curves of CBD after oral administration of CBD + MT (10 + 10) mg/kg and CBD + MT (10 + 40) mg/kg in mice (**B**). CBD: cannabidiol; MT: melatonin.

The plasma concentration–time curves of CBD in mice after the intravenous injection of CBD 2 mg/kg plus oral administration of MT 10 mg/kg are shown in Figure 7A, with the corresponding pharmacokinetic parameters detailed in Table 6. The apparent volume of distribution of CBD (8.491 ± 3.675 L/kg) highly exceeded the plasma volume of the mice, and the clearance (7.04 ± 1.03 L/h/kg) was comparable to the hepatic blood flow in mice. The plasma concentration–time curves of CBD following the oral administration of MT + CBD (10 + 10) mg/kg and MT + CBD (10 + 40) mg/kg are presented in Figure 7B, which shows an approximate 16-fold increase in the AUC of CBD, far surpassing the dose ratio. As per previous literature, non-linear pharmacokinetics of CBD might stem from the saturation of its metabolic enzymes or P-gp transporters, or due to CBD’s inhibitory action on its own metabolic enzymes.

## 4. Discussion

In recent years, studies have confirmed that cannabidiol is a non-psychoactive component of the cannabis plant, which has received widespread attention [27,28,29,30,31]. Due to the druggability issue of melatonin, great effort has been made to address this challenge [19]. In this study, from the perspective of pharmacokinetics, we preliminarily investigates the pharmacokinetic properties of CBD when combined with melatonin in mice. The aim is to demonstrate the in vivo exposure characteristics following the combination of CBD and melatonin, particularly to determine whether CBD influences the in vivo processes of melatonin.

The establishment of a robust method for the quantitative analysis of melatonin and cannabidiol in mouse plasma was the foundation for the aforementioned studies. The LC-MS/MS detection methods for melatonin have been reported in the literature, such as the LC-MS/MS method for quantifying melatonin (MT) in human saliva using liquid-liquid extraction combined with liquid chromatography-tandem mass spectrometry [32]. There are also reports on the LC-MS/MS detection methods for CBD, such as the LC-MS/MS method for extracting and quantifying CBD and its metabolites along with 15 other cannabinoids from 200 µL of human plasma using protein precipitation [33]. Additionally, there is a method for quantifying four cannabinoids, including CBD, in mouse tissues [34], with a sensitivity of 7 ng/mL for CBD. Given that this study is the first to conduct pharmacokinetic research on the combined use of the two drugs, it is necessary to detect both MT and CBD simultaneously. By reviewing the literature and summarizing the conditions of the detection methods for both, we established a method for the simultaneous quantification of MT and CBD in mouse plasma. According to the results of the preliminary experiments, the quantitative linear range for both MT and CBD was 2–1000 ng/mL, with a lower limit of quantification of 2 ng/mL for both. The current method requires only 10 μL of a single plasma sample. After processing the plasma sample with a one-step protein precipitation method, the instrument analysis time is 4 min, which is highly efficient and supports the pharmacokinetic study of a single mouse completing a pharmacokinetic curve.

MT, as an endogenous hormone, has a distinct circadian rhythm in its secretion, with approximately 80% being produced at night. Studies have shown that the endogenous serum concentration of MT ranges from 80 to 120 pg/mL, reaching its peak between 2:00 and 4:00 in the morning, and is extremely low during the daytime, merely amounting to 10 to 20 pg/mL [35]. By employing the LC-MS/MS method established in this study, no MT was detected in the blank plasma samples from all experimental groups of mice. This implies that the endogenous MT concentration in mice does not reach the LLOQ level of this method and does not impact the quantification of exogenous MT.

Exogenous melatonin, although not yet developed as a therapeutic agent, has been the subject of numerous pharmacokinetic studies. In a previous cohort crossover study, volunteers were administrated 10 mg MT either orally or intravenously, resulting in a V_d_ of 1.2 L/kg, a CL of 21.8 mL/min/kg, a t_1/2_ of 39.4 min for intravenous MT, and an oral bioavailability of 2.5% [36]. Another pharmacokinetic study in mice showed that after oral administration of MT at 250 mg/kg, MT peaked rapidly (T_max_ was 10 min), t_1/2_ was 40.91 min, and bioavailability was 29.02% [37]. The findings from the aforementioned studies are largely in agreement with the results of this study. The high apparent volume of distribution of MT in mice suggests its extensive tissue distribution and good membrane permeability. The clearance rate, which is close to the hepatic blood flow rate in mice (5.4 L/h/kg) [38], indicates that MT is primarily metabolized in the liver [39]. After oral administration, absorption is extraordinary rapid, while the bioavailability is only 10%.

In the present study, the impact of co-administered CBD on the pharmacokinetic characteristics of MT is first investigated. To fully observe the effects of CBD on MT, we conducted in vivo experiments on mice that were orally administered MT at a dose of 10 mg/kg alone, and added experiments where different doses of CBD (10 and 40 mg/kg) were co-administered orally, as well as an intravenous dose of CBD (2 mg/kg). The outcomes showed interesting alterations in the blood concentration of MT after CBD was co-administered. Although the time to peak concentration after the oral administration of MT was relatively short, it was essentially the absorption process before reaching the peak. To thoroughly compare the impact of CBD on absorption and metabolism, we artificially divided the MT concentration–time curve into an absorption phase (AUC_(0–0.5h)_) and a distribution and elimination phase (AUC_(0.5–8h)_). According to the results shown in Figure 4 and Table 5, co-administration of a low dose of CBD orally did not change the oral concentration–time curve and pharmacokinetic parameters of MT; however, co-administration of a high dose of CBD orally led to opposite changes in the absorption and distribution/elimination phases of the MT concentration–time curve. The exposure in the absorption phase was reduced to about 50%, while the exposure in the distribution/elimination phase increased to nearly three times the level. Although the overall exposure (AUC_(0–8h)_) remained almost unchanged, the blood concentration of MT became relative stable. Additionally, when CBD was administered intravenously in combination with oral MT, it only increased the exposure in the distribution/elimination phase of MT by about 1.5 times. Based on these observations, we speculate that the impact of oral CBD on MT may involve multiple factors including intestinal absorption and hepatic metabolism. It is known that MT is a substrate for CYP1A2, and CBD is an inhibitor of CYP1A2. Thus, the increased exposure in the distribution/elimination phase of MT is likely mainly due to the inhibitory effect on CYP1A2. The reduction in the absorption phase of the MT concentration–time curve may be due to CBD’s inhibition of the transporters that participate in the intestinal uptake of MT, and the specific mechanism requires further in-depth study.

To further elucidate the characteristics of CBD’s impact on MT, the correlation between the pharmacokinetic parameters derived from the concurrent determination of CBD concentration and MT exposure was analyzed. The results revealed that there was a good positive correlation between CBD plasma exposure and MT distribution/elimination phase exposure across various groups. On the other hand, the C_max_ of CBD negatively correlated with the C_max_ of MT. These findings suggest that CBD exerts a complex concentration-dependent inhibitory effect on MT metabolism and absorption.

The current research, although it is the first to discover that CBD has an impact on the pharmacokinetics of MT, still encompasses obvious limitations. Not introducing metabolites of CBD and MT in the current determination impacts an in-depth understanding the interaction between MT and CBD. The DDI of CBD toward MT requires further verification in in vitro models based on the strength of cytochrome P450 (CYP) enzymes, especially CYP1A2 inhibition, and its mechanism. Additionally, the inhibition based on intestinal uptake transporters should also be evaluated to further confirm the in vivo DDI results. Finally, due to species barrier issues, the DDI results of CBD and MT in mice may not necessarily characterize the human DDI results. Although the interspecies differences in CYP1A2 are relatively small and it is expressed in all species of animals, there are still differences in activity [40]. We have preliminarily verified using in vitro experiments that CBD also has a significant inhibitory effect on MT in human liver microsomes (unpublished data). The prospects of CBD co-administration with MT in humans require in-depth research.

In conclusion, this study successfully established and validated an LC-MS/MS quantitative analysis method for the simultaneous detection of CBD and MT in mouse plasma, marking the first time such a method has been developed. Utilizing this method, it was discovered for the first time in mice that the co-administration of CBD exerts certain effects on the absorption and clearance of MT in vivo. Although the overall exposure of MT was not significantly affected after co-administration of CBD, the more stable blood concentration would be beneficial for the exertion of MT’s therapeutic effect. This research offers a scientific foundation for further exploration into the combined therapeutic potential of CBD and MT.

## Figures and Tables

**Table 1:** Accuracy and precision of LC-MS/MS methods for CBD and MT in mice plasma.

Analytes	Concentration (ng/mL)	Intra-Day (*n* = 6)						Inter-Day (*n* = 18)	
		Accuracy (%)			RSD (%)			Accuracy (%)	RSD (%)
CBD	2	111.17 ± 4.77	111.82 ± 3.84	113.37 ± 5.18	4.3	3.4	4.6	112.12 ± 4.45	4.0
	4	94.38 ± 5.43	101.62 ± 3.33	101.18 ± 4.16	5.8	3.3	4.1	99.06 ± 5.35	5.4
	80	98.85 ± 1.07	97.83 ± 1.72	98.42 ± 2.28	1.1	1.7	2.3	98.37 ± 1.71	1.7
	800	112.52 ± 1.20	110.65 ± 1.86	112.00 ± 3.24	1.1	1.7	2.9	111.72 ± 2.28	2.0
MT	2	99.23 ± 5.16	103.30 ± 6.10	87.55 ± 4.16	5.2	5.9	4.7	96.69 ± 8.43	8.7
	4	97.83 ± 3.28	102.33 ± 2.21	94.08 ± 4.33	3.3	2.1	4.6	98.08 ± 4.71	4.8
	80	100.60 ± 1.15	100.25 ± 1.00	99.22 ± 1.12	1.1	1.0	1.1	100.02 ± 1.19	1.2
	800	101.85 ± 0.67	101.45 ± 0.77	101.22 ± 1.76	0.7	0.7	1.7	101.51 ± 1.14	1.1

CBD: cannabidiol; MT: melatonin.

**Table 2:** Method recoveries and matrix effects of CBD, MT, and MT-D4 in mice plasma (mean ± SD, *n* = 6).

Analytes	Concentration (ng/mL)	Extraction Recovery (%)		Matrix Effect (%)	
		Mean ± SD	RSD	Mean ± SD	RSD
CBD	4	93.98 ± 4.27	5.0	109.94 ± 4.70	4.7
	80	95.51 ± 2.42	2.8	/	/
	800	90.43 ± 1.86	2.3	102.29 ± 2.27	2.4
MT	4	95.63 ± 5.64	6.5	99.21 ± 3.81	4.2
	80	98.85 ± 3.20	3.5	/	/
	800	93.76 ± 1.45	1.7	100.93 ± 1.69	1.8
MT-D4 (IS)	10	97.29 ± 3.05	3.4	99.86 ± 1.90	2.1

/: Not available. CBD: cannabidiol; MT: melatonin; MT-D4: melatonin-D4; IS: internal standard.

**Table 3:** Stability of CBD and MT in mice plasma (*n* = 3).

Conditions	Values	CBD			MT		
		4 ng/mL	80 ng/mL	800 ng/mL	4 ng/mL	80 ng/mL	800 ng/mL
RT for 4 h	Accuracy (%)	102.57 ± 7.13	94.77 ± 4.16	102.60 ± 1.00	96.43 ± 2.80	99.97 ± 3.36	97.33 ± 0.68
	RSD (%)	7.0	4.4	1.0	2.9	3.4	0.7
4 °C for 24 h	Accuracy (%)	92.07 ± 4.02	91.13 ± 2.19	104.43 ± 1.21	92.70 ± 1.48	99.03 ± 1.88	100.53 ± 1.06
	RSD (%)	4.3	2.4	1.1	1.6	1.9	1.1
Freeze–thaw three times	Accuracy (%)	96.97 ± 6.81	91.23 ± 1.10	103.13 ± 1.60	95.47 ± 3.18	95.97 ± 0.51	97.93 ± 2.42
	RSD (%)	7.0	1.2	1.5	3.3	0.6	2.5
In auto-sampler for 24 h	Accuracy (%)	110.77 ± 4.82	99.20 ± 2.08	112.63 ± 1.89	99.87 ± 2.81	101.20 ± 2.14	101.50 ± 0.36
	RSD (%)	4.3	2.1	1.7	2.8	2.1	0.3
−40 °C for 7 d	Accuracy (%)	99.07 ± 14.15	90.07 ± 5.71	106.33 ± 6.14	88.67 ± 5.09	95.63 ± 3.63	98.13 ± 1.64
	RSD (%)	14.3	6.4	5.8	5.7	3.8	1.7
−40 °C for 21 d	Accuracy (%)	95.13 ± 10.59	90.33 ± 5.38	108.23 ± 5.22	89.40 ± 2.78	93.63 ± 2.48	97.83 ± 0.75
	RSD (%)	11.1	5.9	4.8	3.1	2.6	0.7

RT: Room temperature; CBD: cannabidiol; MT: melatonin.

**Table 4:** Dilution accuracy of CBD and MT in mice plasma (*n* = 6).

Analyte	Concentration (ng/mL)	Dilution Factor	Accuracy (%)	RSD (%)
CBD	5000	100	−6.0	2.2
MT	5000	100	0.6	7.9

CBD: cannabidiol; MT: melatonin.

**Table 6:** Pharmacokinetic parameters of CBD after administration of CBD in mice (mean ± SD, *n* = 6).

Parameters	Unit	*i.v.* (mg/kg)	*p.o.* (mg/kg)	
		MT 10 *p.o.* + CBD 2 *i.v.*	MT + CBD (10 + 10)	MT + CBD (10 + 40)
t_1/2_	h	1.53 ± 0.11	1.05 ± 0.09	1.65 ± 0.05
T_max_	h	/	0.42 ± 0.20	0.63 ± 0.26
C_max_	ng/mL	/	103.52 ± 25.67	1820.4 ± 823.7
AUC_(0–8h)_	h × ng/mL	279.36 ± 41.69	134.80 ± 28.69	2156.5 ± 574.5
AUC_(0–∞)_	h × ng/mL	288.58 ± 38.60	138.12 ± 29.05	2186.8 ± 578.2
MRT_(0–t)_	h	0.79 ± 0.10	1.04 ± 0.13	1.53 ± 0.11
V_z_	L/kg	27.9 ± 17.9	/	/
V_ss_	L/kg	8.49 ± 3.67	/	/
CL	L/h/kg	7.04 ± 1.03	/	/

/: Not available. CBD: cannabidiol; MT: melatonin.

## Data Availability

The original contributions presented in the study are included in the article, further inquiries can be directed to the corresponding author.
